# 

**Published:** 1908-04-15

**Authors:** 


CONTENTS

Event and Comment - - - - - - - - - 163
Dental Remedies, - *By Dr. J. P. Buckley, Ph.G., D.D.S.* 168
Drugs; Their Uses, Abuses and Measurements,
*By David Stern, B.S., D.D.S.* 185
Spinal Anesthesia from the Standpoint of the Patient,
*By John S. Marshall, M.D.* 193
Obituary - - - - - - - - - - - 199
Indents - - - - - - - - - - - 201
Announcements - - - - - - - - - - 212

